# Experimental Global Warming Potential-Weighted Specific Stiffness Comparison among Different Natural and Synthetic Fibers in a Composite Component Manufactured by Tailored Fiber Placement

**DOI:** 10.3390/polym16060726

**Published:** 2024-03-07

**Authors:** Gustavo de Abreu Cáceres, Tales de Vargas Lisbôa, Cindy Elschner, Axel Spickenheuer

**Affiliations:** 1 Leibniz-Institut für Polymerforschung Dresden e. V., 01069 Dresden, Germany; gustavo.abreu@ufrgs.br (G.d.A.C.); elschner@ipfdd.de (C.E.); spickenheuer@ipfdd.de (A.S.); 2 Department of Mechanical Engineering, Federal University of Rio Grande do Sul, Porto Alegre 90010-150, Brazil

**Keywords:** natural fibers, tailored fiber placement, carbon footprint, technical natural fibers, mechanical performance

## Abstract

This work aims to evaluate experimentally different fibers and resins in a topologically optimized composite component. The selected ones are made of carbon, glass, basalt, flax, hemp, and jute fibers. Tailored Fiber Placement (TFP) was used to manufacture the textile preforms, which were infused with two different epoxy resins: a partly biogenic and a fully petro-based one. The main objective is to evaluate and compare the absolute and specific mechanical performance of synthetic and natural fibers within a component framework as a base for improving assessments of sustainable endless-fiber reinforced composite material. Furthermore, manufacturing aspects regarding the different fibers are also considered in this work. In assessing the efficiency of the fiber-matrix systems, both the specific stiffness and the specific stiffness relative to carbon dioxide equivalents (${\mathrm{CO}}_{2}^{\mathrm{eq}.}$) as measures for the global warming potential (GWP) are taken into account for comparison. The primary findings indicate that basalt and flax fibers outperform carbon fibers notably in terms of specific stiffness weighted by ${\mathrm{CO}}_{2}^{\mathrm{eq}.}$. Additionally, the selection of epoxy resin significantly influences the assessment of sustainable fiber-plastic composites.

## 1. Introduction

Carbon fibers are often used as reinforcements in plastics due to their exceptional mechanical properties. However, the processes for producing these fibers alone have a high global warming potential (GWP) compared to other reinforcing fibers [1,2]. In particular, natural fibers have a much lower environmental footprint; however, they are used much less frequently in fiber-plastic composites. They are, unfortunately, not used as reinforcement when structural performance is crucial for several reasons: problems in fiber-resin interface, lower strength properties, moisture absorption, higher variance in performance, etc. [3,4,5,6]. Still, some natural fibers can achieve mechanical performances (stiffness) comparable to glass fibers [7,8].

Multiple studies have investigated the environmental impacts of fiber-reinforced plastics using life cycle analysis (LCA), which comprehensively quantifies potential environmental effects throughout a product’s life cycle—with contributions from raw material acquisition, manufacturing, distribution, and use, to end-of-life product disposal. This analysis accounts for various environmental consequences, health impacts, and risks related to resource usage [9]. However, there is a lack of homogeneity in the application of the LCA method. The definition of methodological approaches retains an element of subjectivity, a factor seldom addressed when interpreting results [10]. In addition, the life cycle stages covered (e.g., with or without the use phase or with or without consideration of the production and procurement of raw materials) varies enormously in the available publications [11]. Duflou et al. [12] presented an LCA study based on three impact measures (cumulative energy demand, greenhouse gas emissions, and environmental impact scores) for bio-based fiber-reinforced polymer composites as an alternative to metallic structures. Comparisons were made with, among other materials, glass fiber-reinforced polymers × aluminum and carbon fiber-reinforced polymers (CFRP) × steel. With respect to the first, the LCA analysis describes how aluminum structures might perform better since they produce slightly lighter structures and are easily recyclable. For the latter, the LCA suggests that steel and aluminum are eco-friendlier after long usage of the components: 132,000 km for steel automotive panels and 70,000 km for aluminum aircraft applications. Khorgade et al. [13] focused on a GWP analysis covering the cradle-to-gate period of two bridges containing both steel and CFRP reinforcements. They have obtained a favorable ${\mathrm{CO}}_{2}$ emission for the carbon counterpart given the fact that both the amount of concrete and reinforcement are to be used in the CFRP configuration. Mindermann et al. [7] have produced a deep and interesting investigation of the sustainability and structural performance of natural fibers when used in coreless filament winding. Firstly, the authors provided an important set of fiber and resin properties, including composition, mechanical behavior, GWP, and price. They have selected 12 different fibers and evaluated them for both manufacturing aspects and structural behavior, using four-point bending tests. Results have shown that flax fibers performed better when compared to other bio-based fibers. For most LCA impact categories, in refs. [7,14,15] it was also exhibited that the production of basalt fibers causes less environmental impact than the production of glass fibers. Considering the natural fibers alone, Beus et al. [16] have covered several (plant-based) fiber types and different transport configurations—from the plantations to the industry—as well as cultivation procedures.

As suggested by Mindermann et al. [7], it is worth noticing that the resin system plays a significant role in the composite ecological performance, mainly when natural fibers are taken into account. Niutta et al. [17] have compared quasi-static mechanical properties of petroleum- and bio-based epoxy resins and found no significant difference between them, apart from the GWP, which gives the designer the freedom to choose a bio-based epoxy system not only for ecological but also for technical reasons. La Rosa et al. [11] have introduced an LCA comparison study between a bio-based polymer composite and a conventional glass/epoxy one. Their results have shown that the major impact, in terms of environmental impact, is given by the resin system.

With regard to manufacturing processes capable of dealing with diverse fiber characteristics, tailored fiber placement (TFP) technology [18,19,20,21] can be cited. This textile manufacturing technique can deal with many fibrous materials by embroidery means. Little to no modifications are required when changing fiber types. Furthermore, by using TFP, the designer has a larger degree of freedom in terms of fiber placement: the rovings are stitched over a base material in predefined paths with high precision. Variations of properties due to the stitching process are evaluated by refs. [22]. Almeida Jr. et al. [23] have improved an already optimized component and results have shown that outstanding mechanical behavior can be obtained by TFP-made components even when compared with other high-performance composites. Poniecka et al. [24] have used the process to evaluate the mechanical properties of different configurations of flax fibers—from UD to woven fabric—considering different TFP parameters. Their results have shown that strength values were higher in UD TFP samples when compared to conventional UD-reinforced composites. Furthermore, the obtained elastic modulus of flax fibers was larger than that of glass fibers.

Many studies have considered comparisons in terms of evaluating simple tests on coupons [3,4,5,7]. To the authors’ knowledge, little to none has been performed in terms of a component level. The objective of this work is to apply six different fibers, both natural and synthetic, as reinforcement in a component originally optimized for CFRP [20,23], and study manufacturing aspects and mechanical performance along with the GWP. The importance of comparing performances through a composite component (not coupons) lies in consideration of the manufacturing procedures, possible adaptations required for natural fibers and biogenic epoxy, and their influence on the component’s mechanical response. The fibers chosen are carbon, E-glass, basalt, flax, hemp, and jute. Furthermore, two resin systems were included in the experiment: a petroleum and a bio-based resin. The latter is 36% plant-based [25], reducing its GWP. Firstly, basic experiments, such as linear density, density of the resins, and dry fiber tensile stiffness are performed to compare some of the mechanical properties of the composite constituents. Then, the topology-optimized component is manufactured and tested. Key results have shown that flax fibers have the potential to substitute glass fibers, for example, if one considers both mechanical properties and GWP. Moreover, basalt fibers have shown an outstanding performance. Furthermore, the resin nature plays little to no influence when carbon fibers are considered whereas it is the major aspect of GWP for plant-based natural fibers.

## 2. Configuration and Properties, Specimens Preparation, and Material Tests

### 2.1. Tailored Fiber Placement

**Figure 1:**
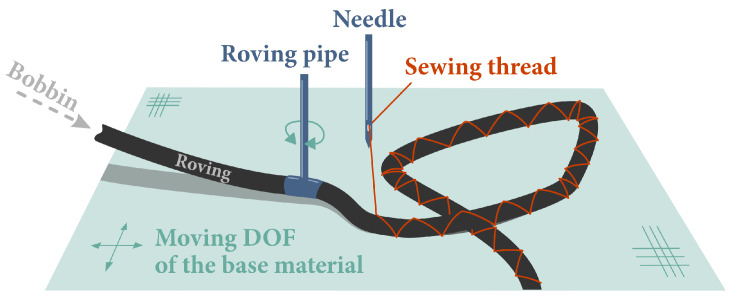
Scheme of the TFP process [26].

Tailored Fiber Placement (TFP) is an embroidery-based technique developed in the Leibniz-Institut für Polymerforschung Dresden e.V. (IPF) that allows the production of textile preforms with high precision and with a higher degree of freedom (when compared to other methods) regarding fiber orientation and local thickness (see Figure 1). Using a rotating pipe/delivery eye, endless fibers are stitched in a predefined orientation onto a base material, which is fixed in a moving frame. By placing rovings together or apart, one also controls the local thickness, increasing or reducing it, respectively. Furthermore, any fibrous material that can be made as a roving could, in principle, be applied in this manufacturing process. In the same sense, the base material can also be made of different materials and constructions, from woven fabrics—made of virtually any fibrous material—to paper. More information about the process can be found in refs. [18,20,21].

An important feature of the TFP process concerning material usage during the manufacturing of components is the neat-shape preforming: the volume of fibers and resin used in a component is close to the final part. Little trimming of preforms is required, for example, saving not only costs but also reducing the waste during the manufacturing stages, which means also a reduction of the environmental impact regarding the waste at a component level. Furthermore, the post-processing step is mostly reduced to grinding some areas, if it is required at all.

### 2.2. Fibers and Resins

The fibers considered in this work are presented in Table 1. The cross-section is defined through their density and their linear density (obtained through datasheets from the manufacturers). The roving’s cross-section is of great importance given the fact that the thickness profile of the component will change depending on the fineness of the applied fibers. The manufacturers of the fibers are Teijin Carbon Europe GmbH (Wuppertal, Germany), Lange+Ritter GmbH (Gerlingen, Germany), Deutsche Basalt Faser GmbH (Sangenhausen, Germany), Safilin (Sailly-sur-La-Lys, France), Kanirope, and Kanirope (Dortmund, Germany) for carbon, glass, basalt, flax, hemp, and jute, respectively. Since the natural fibers based on plants are hydrophilic, these rovings were kept dry during the entire process of infusion and the produced components were kept below 50% humidity. It is important to mention that the values shown in Table 1 are extracted from manufacturer datasheets. In Section 3.1, tests will be performed to verify these values.

Two different epoxy resins are used along with the six fibers shown in Table 1: InfuGreen 810 (epoxy) + SD8822 (hardener) from Sicomin Epoxy Systems and L20 (epoxy) + EpH161 (hardener) from Westlake Epoxy, InfuGreen 810 + SD 8822 (Green Epoxy) has 29% ± 3% of plant-based components [25]. Properties of both resins are presented in Table 2. The curing (and post-curing) processes are slightly different: InfuGreen 810 + SD 8822 was cured at 40 °C for 3 h and post-cured at 40 °C for 8 h, and L20 + EpH 161 was cured at approx. RT for 1.5 h and post-cured at 60 °C for 10 h.

Different impact categories can be considered in an LCA analysis [11,13,14]. Here, we focus only on the global warming potential (GWP), which defines the amount of equivalent ${\mathrm{CO}}_{2}$ emitted during the process of manufacturing the fibers. We have not taken into account either the service life of the composite components or their end-of-life scenarios. It is well known from the literature that these values can vary due to the methodology and the region where they are calculated. Some works [7,12,13,16] have introduced a summary of GWP data. These, along with others, are presented in Table 3 for the six fibers considered here, and are to be used in the evaluation of the GWP of each component. It is worth noting that the GWP of carbon fiber is ten times larger than the second largest GWP, i.e., that of glass. However, CFRPs are known to have excellent stiffness-to-weight properties. A proper comparison, that includes both concepts, is required and sought in this paper. Furthermore, as observed in the literature, these values can strongly vary. The chosen ones, when a larger dataset was available, were related to Germany- or Europe-based studies, since energy-carbon-related variables would then match more closely the regions where the research was conducted.

As stated by the manufacturer, the bio-based epoxy from Sicomin has, on average, 40% less GWP than the petroleum-based ones [31]. However, the values used for the bio-based epoxy are from SuperSap (from Entropy Resins) [11], which is within the limit suggested by the manufacturer Sicomin, since no reliable data from InfuGreen 810 were found by the authors. Table 4 introduced the data found regarding the GWP of the epoxy resins while Table 5 summarizes the values considered within this work.

### 2.3. Manufacture of the Specimens—The Brake Booster

**Figure 2:**
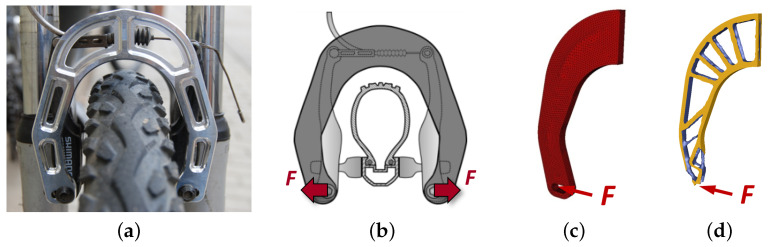
Brake booster (BB) where (**a**) presents an aluminum BB on a bike, (**b**) represents the optimization design space along with the boundary conditions (forces applied to the component), (**c**) shows the (symmetric) finite element model, and (**d**) exposes the topologically optimized result [20].

**Figure 3:**
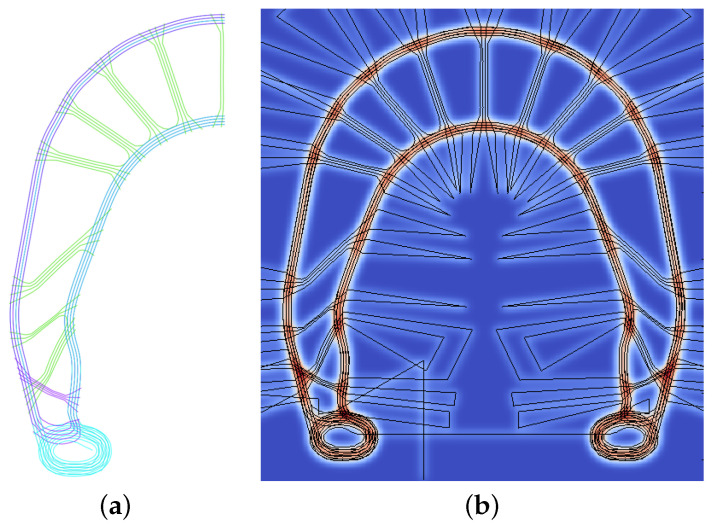
Position of the rovings where (**a**) presents results from the optimization (extracted from ref. [23]), and (**b**) shows the pattern of the rovings along the thickness distribution (which changes depending on the fiber’s type and local fiber path density).

The brake booster (BB) (Figure 2a), is a component that has been used as a demonstrator of the TFP technology due to its high optimization level [18,20]. The BB is a component attached to a bicycle brake to improve its performance. Initially, a topology optimization (Figure 2b–d) based on an isotropic material was performed and, later, a genetic optimization concerning the number of rovings in each truss-like arm [20,23] was conducted. Compared to a quasi-isotropic CFRP-made brake booster a +330% larger stiffness was obtained [23]. The results of the rovings placement and local thickness are shown in Figure 3a and Figure 3b, respectively. This component is considered here due to the exploited use of endless fiber characteristics.

**Figure 4:**
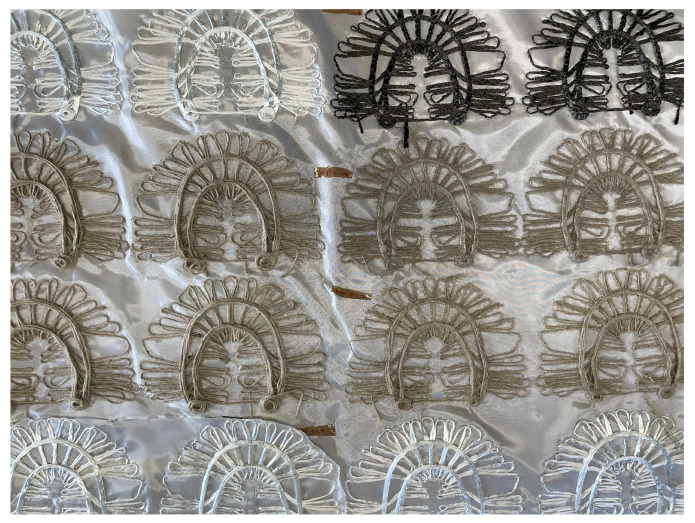
Preforms manufactured by TFP and made of different roving materials: glass, basalt, flax, and jute.

The information in Figure 3a—the rovings’ center lines—is used as input for the software EDOpath from Complex Structures GmbH, which creates the CNC-code for the TFP machines. Figure 4 shows several of the preforms made of different materials still uncut from the base material. These are precisely cut and arranged in pairs, aiming to achieve symmetry, with the base material positioned between them. After that, the cut preforms are inserted into molds for composite consolidation. The used base material in all specimens is a glass-fiber woven fabric (102 $g/{\mathrm{cm}}^{2}$).

The molds’ construction follows a procedure described in ref. [34] referred to as a “low-cost rapid prototyping process”. One property of the TFP technology, in terms of manufacturing components, is the local thickness variation. In order to take full advantage of this degree of freedom, in terms of structural behavior, the consolidation molds should follow, precisely, this variation so as to avoid creating resin-rich zones or areas with varying average fiber volume content. A straightforward method would be the manufacturing of metallic molds, however, this step is time-consuming and also expensive.

**Figure 5:**
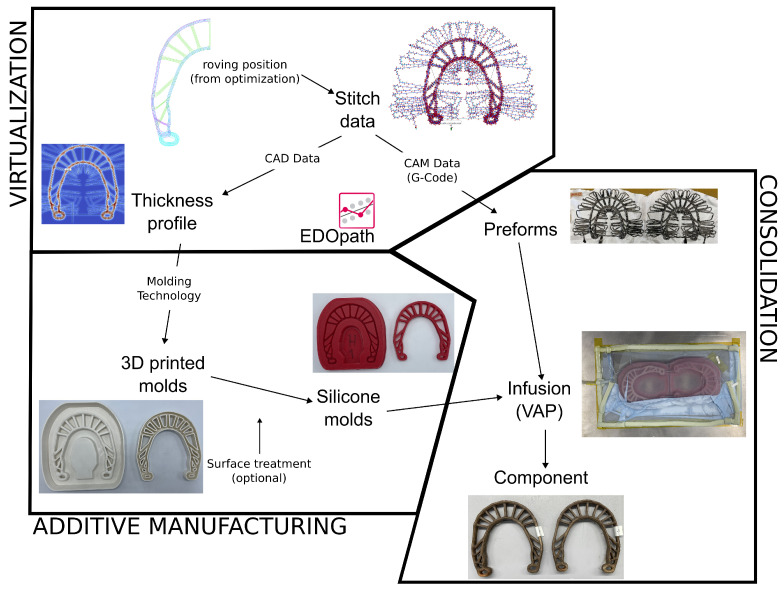
Low-cost rapid prototyping: flowchart of how the specimen is manufactured.

This is essentially the motivation for developing the idea of silicone molds, which are based on additive manufacturing processes. A flowchart (Figure 5) presents the scheme of producing molds in this fashion. Firstly, the stitch data is created, having as input the central position of the rovings as well as its properties. Then, two steps are necessary: the development of the molds and the consolidation itself. From the thickness profile of the component, which is provided by EDOpath software (version 2.0) [21], its positive shape is 3D-printed in two forms: bottom and top casting molds. After that, silicone is poured into these molds, creating the consolidation molds. Inside these molds, the preforms are placed and the infusion is performed. It is important to mention that all surface characteristics of the molds’ surface are transferred to the silicone molds (see Figure 5—surface treatment). For that reason, one uses ABS (or similar) and acetone for smoothing the surface, for example.

Silicone molds are not only more cost-effective [34] but also easier to handle, facilitating the positioning of the preform inside the molds and the demolding process due to their flexibility. Geometric tolerances are achieved given the vacuum assisted process (VAP) used for the consolidation; it forces both parts of the molds to be fixed together. If the mold is well designed, the difference between the virtual and real thicknesses is less than 5%, even for thicker parts. Furthermore, these silicone molds can be used 5–10 times when considering the petroleum-based epoxy. After that, the silicone starts becoming stiffer, up to the point where damage, such as cracks, are visible. Qualitatively, the bio-epoxy will be tested so as to ascertain whether it produces more or less damage in the mold.

### 2.4. Experiments

Some experiments were performed on the fibers and resin as well as on the composite part as follows.

**Figure 6:**
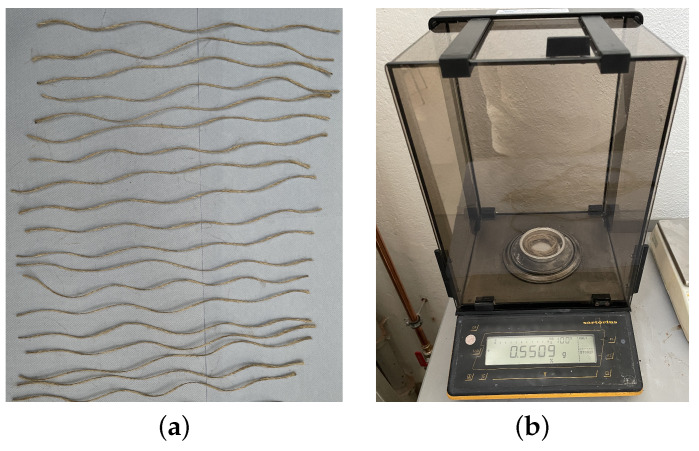
(**a**) Specimens and (**b**) measurement by analytical weighting scale for fiber fineness.

**Figure 7:**
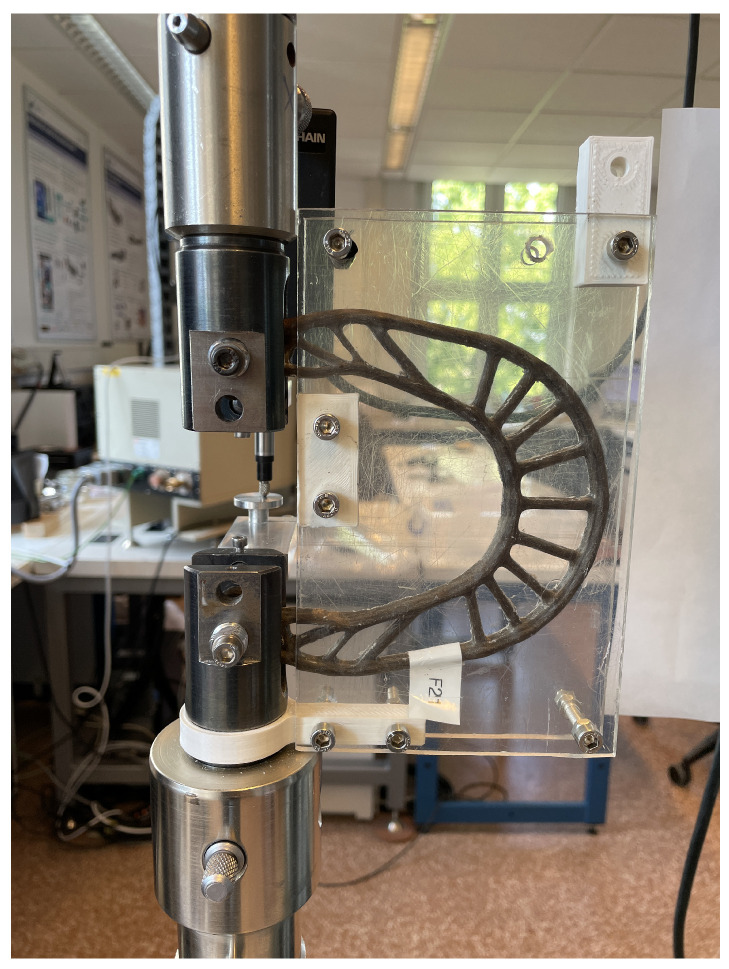
Brake booster tensile test (flax-BB consolidated with PEp).

- Fibers—fiber fineness: A finesse evaluation and a simple tensile test with the rovings were made. For the tex number, 20 specimens with 0.5 m of each fiber type were measured as shown in Figure 6. With the tex number, one can correct the roving cross-section and, thus, the molds (thickness). An analytical weighting scale was used to perform the measurements.
- Fibers—tensile test: With the roving tensile tests performed using ISO 3341 [35]/DIN EN ISO 2062 [36] standards, the stiffness/tex can be evaluated for comparisons among the fibers since the literature presents different values for fiber stiffness, mainly for the natural mineral- and plant-based fibers [7]. Five 0.5 m length specimens for each fiber type were considered for this test. All tests were performed in a controlled environment (23 ± 2 °C with 50% relative humidity).
- Resin—density: the density is evaluated considering the norm DIN EN ISO 1183 [37]. A for a proper definition of their GWP within the component. Five specimens considering each resin type were tested.
- Composite—tensile test: The composite parts are tested in a similar way to their use. Figure 7 shows the test. Acrylic layers on both sides of the specimen were attached. This is made to avoid premature buckling under high loading caused by small constructive asymmetries. For each material and resin, five samples were tested (60 brake boosters in total). The experimental parameters were: load cell 10 kN, preload: 50 N, and load speed: 10 mm/min. All tests were performed in a controlled environment (23 ± 2 °C with 50% relative humidity).

### 2.5. Specific Stiffness and GWP-Weighted Specific Stiffness

The specific stiffness is an important property, mainly for the aerospace industry, as it combines the stiffness of the component/material with its mass. The definition, applied to the case of the BB, is straightforward and it is as follows

(1) $$\rho_{s}=\frac{k}{m^{BB}}=\frac{k}{m_{f}^{BB}+m_{r}^{BB}}\;\;,$$

where $\rho_{s}$ corresponds to the specific stiffness, *k* is the measured (or absolute) stiffness, and $m^{BB}$, $m_{f}^{BB}$, and $m_{r}^{BB}$ denote the brake booster *total* mass, *fiber* mass, and *matrix* mass, respectively. The measured stiffness, *k*, is defined as

(2) $$k=\frac{F^{j}-F^{i}}{u^{j}-u^{i}}$$

where *F* and *u* represent the force and displacement, respectively, of the BBs tensile test at *i* and *j* positions. From the specific stiffness (Equation (1)), one can develop a GWP-weighted specific stiffness in a very straightforward way: multiplying each mass by their particular GWP as follows

(3) $$\frac{\rho_{s}}{\mathrm{kg}\;{\mathrm{CO}}_{2}^{\mathrm{eq}.}}=\frac{k}{m_{f}^{BB}\;{\mathrm{GWP}}_{f}+m_{r}^{BB}\;{\mathrm{GWP}}_{m}}$$

in which ${\mathrm{GWP}}_{f}$ and ${\mathrm{GWP}}_{m}$ represent the fiber and matrix GWPs, respectively. It is worth noticing that the idea Equation (3) can be used with almost all LCA parameters since they generally consider a ratio between a quantity (usually a toxic/dangerous substance or effect) per mass of material produced. This is relatively similar to the specific stiffness in nature. Equations (1) and (3) are to be used in the comparison of the mechanical behavior and the (weighted)-carbon footprint of each aforementioned fiber+resin systems.

## 3. Results

### 3.1. Fiber and Resin Tests

First, the fineness of the fibers was measured; the corrected thickness of the roving is presented in Table 6. It is observed that all natural plant-based fibers had lower finenesses than defined by the manufacturers. The largest difference was with hemp fibers, where the measured value was 15% smaller (see Table 1). Furthermore, the variation was much greater than the synthetic fibers and the basalt fiber (natural but mineral-based).

**Figure 8:**
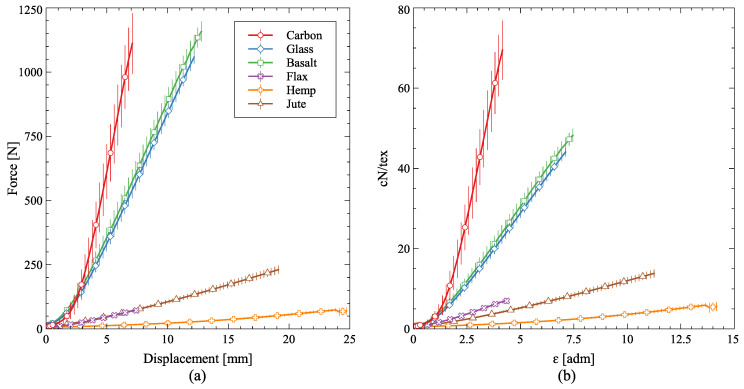
(**a**) Force × displacement and (**b**) force/fineness × strain of the dry fibers. Vertical bars have a total length of two standard deviations.

Figure 8 presents the force × displacement and force/fineness × strain of the six dry fibers along with the experimental errors. With regard to the force × displacement, the errors are relatively small, even in the case of natural fibers. However, given the propagation of uncertainties, the errors of the force/tex × strain curves for the natural fibers are much larger given their deviations (see Table 6 of fineness).

Carbon fibers are the stiffest of the chosen set, followed by basalt and glass (both very close to each other) in both stiffness and strength. Flax and jute have also shown similar behavior, with differences when weighted by the fineness (flax slightly stiffer) and elongation (jute much larger). Also, jute gave the largest force/fineness to strength ratio of the natural fibers. Hemp presented the worst mechanical behavior of the fiber set, although it had the largest elongation, which is also described in Ref. [7].

The density of the resins was also experimentally evaluated and Table 7 shows the obtained values and the differences to the manufacturer datasheets. Small discrepancies between the test results and the manufacturer data were found. Moreover, little to no difference was obtained between the petroleum- and bio-based epoxies, with respect to their densities.

### 3.2. Adaptation to the Manufacturing Process

One of the objectives of this work was to evaluate and compare the low-cost rapid-prototyping process (see Figure 5) for natural fibers. Despite the preforms being manufactured without any particular modification to the process/machine, their placement inside the molds was more laborious than carbon, glass, and basalt fibers. Furthermore, the thickness variation by placing natural plant-based rovings together or apart does not follow the same behavior as the aforementioned fibers. Usually, the stitching yarn compresses the roving, distributing laterally the rovings’ cross-section, thus reducing the thickness, which was not observed for plant-based natural fibers. For this reason, larger thicknesses of the dry preforms than expected were obtained during the manufacturing of the specimens.

Corroborating this qualitative evaluation, Table 8 shows the mass and fiber volume content ($f_{vc}$) of the specimens. To obtain the mass values of the dry preforms, the molds were weighted with and without the preforms. Then, after the infusion, the components were weighted again, to obtain the mass of the resin. Since the molds fix the lateral/planar dimensions, the smaller $f_{vc}$ obtained in the natural fibers was a result of the larger thicknesses. As aforementioned, the manufacturing process achieves $f_{vc}$ in between 50% and 55%.

It is observable that the $f_{vc}$ from carbon, glass, and basalt fibers stayed around 50% with the last having an average of around 48%. The largest $f_{vc}$ from plant-based natural fibers is around 43% obtained by the flax fibers, while the smallest is from hemp fibers, closely followed by the jute fibers. Qualitatively, BBs made of jute were the most complex to manufacture due to the aforementioned laborious work of placing the preforms inside the silicone molds. Moreover, no improvement nor faster deterioration of the molds is observed for the molds used in the bio-epoxy infusions.

Another particularity of preforms stitched with rovings made of natural fiber is the possibility of local damage due to inherent properties of the manufacturing procedure, as pointed out by ref. [38]. As is well known, during the embroidery process, a stitching point is usually performed in the roving, generating the so-called eye-like structure [22]. Fibers such as carbon and glass, do not show damage.

### 3.3. Brake Booster Tests

**Figure 9:**
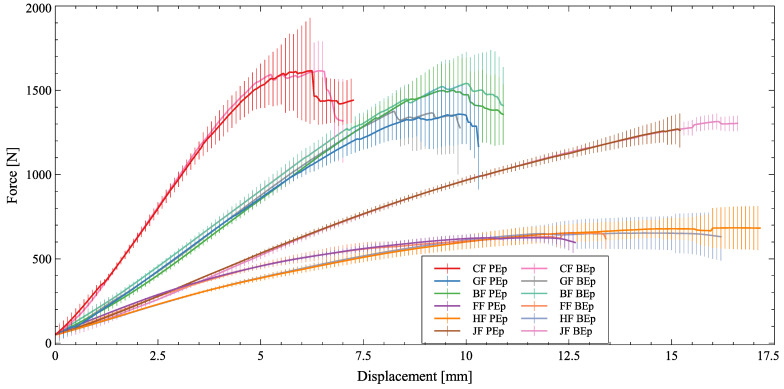
Force × displacement of the brake boosters. Vertical bars have a total length of two standard deviations.

The results from the BB tests are introduced in Figure 9. The obtained results are similar to the dry fiber test (Figure 8): carbon with the highest stiffness, followed by basalt and glass, close together. Then, the plant-based natural fibers appear with similar stiffness but different elongations and strengths. Jute fibers show the largest strength among the plant-based natural fibers, slightly below glass and basalt. One should, however, observe that jute specimens are the heaviest ones among all fiber+resin systems tested. The behavior of glass and basalt fibers was similar, with the basalt having a slightly larger strength. Hemp and flax fibers performed the worst, although the first one had the largest elongation. However, as aforementioned, these components are lighter than the jute ones. Still, at the beginning of the deformation process, their stiffnesses are nearly the same when one compares only the plant-based natural fibers.

Despite being bending test responses, these results were different from Ref. [7]: there, flax fibers performed better in terms of stiffness than jute ones. In contrast, the large elongation behavior of hemp fibers was also observed in that study. Furthermore, no significant difference was observable between the mechanical behavior with respect to the two different epoxies, with similar results to Ref. [17], now considering a composite part.

### 3.4. Specific Stiffness and Specific Stiffness Carbon Footprint

**Figure 10:**
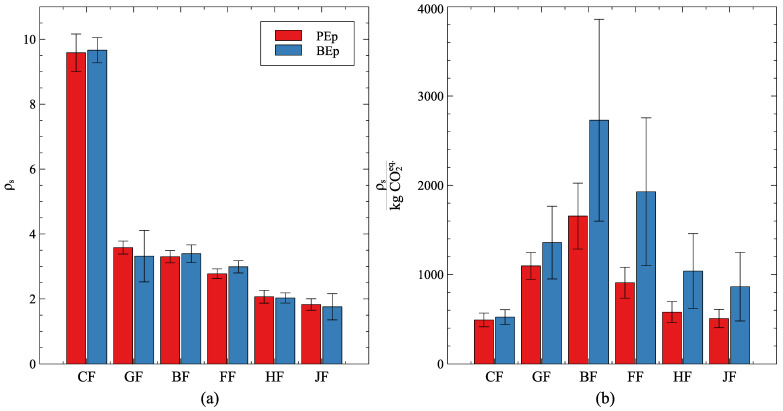
(**a**) Specific stiffness of the evaluated fibers and (**b**) GWP-weighted specific stiffness. Vertical bars have a total length of two standard deviations.

The stiffness (Equation (1)) of all specimens is calculated between 2.0 mm and 2.5 mm displacement. Figure 10a introduces the comparison between the obtained results. Again, similarly to the dry fibers, the carbon-BB stiffness is around three times larger than the glass- or basalt-BB. As observed by other studies [7,24], the behavior of the flax fibers, in terms of stiffness, is close to both basalt and glass fibers. It is important to mention, that the average $f_{vc}$ obtained in the specimens is below the intended one. In other words, the performance of flax fibers could be improved even further.

Figure 10b presents the GWP-weighted specific stiffness (Equation (3)). One might notice that the error bars should not be seen only as deviations in the analysis. As presented in Table 5, the interval of variation from the GWP data can be large. For example: basalt fibers and bio-based epoxy have, in this study, GWP variations around 70% and 50% of their average value, respectively. This is the reason, for example, that the BF+BEp has around 41% of deviation.

Interesting results are observed in Figure 10b. The performance of carbon fiber material, regardless of the epoxy system, is closer to hemp and jute with PEp as resin. This leads to the next conclusion drawn from the graph: natural fibers profit from the use of BEp in terms of GWP-weighted specific stiffness much more than synthetic fibers. In the case of flax, more than double is obtained (on average). Furthermore, basalt and flax were the ones with the best-weighted stiffness among the tested fibers. Curiously, considering PEp alone, carbon fiber got better results than hemp and jute. However, even with three times larger specific stiffness, CF has a (much) lower performance when compared with hemp and jute with BEp.

**Figure 11:**
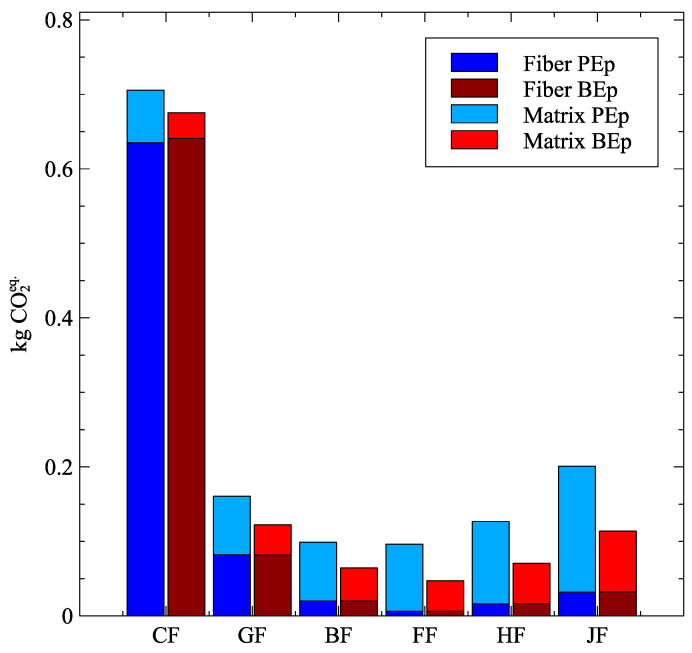
Stacked kg ${\mathrm{CO}}_{2}^{\mathrm{eq}.}$ of fiber and matrix for each fiber-matrix system.

So, to further corroborate the results obtained in Figure 10, the emissions were broken into fiber and resin contributions and introduced in Figure 11. As one can see, the majority of CFRP emissions correspond to fiber emissions. In the opposite direction, the emissions of plant-based natural fibers are essentially resultant from the resin, even considering BEp. The emissions of the GF are mixed between fibers and resins.

**Figure 12:**
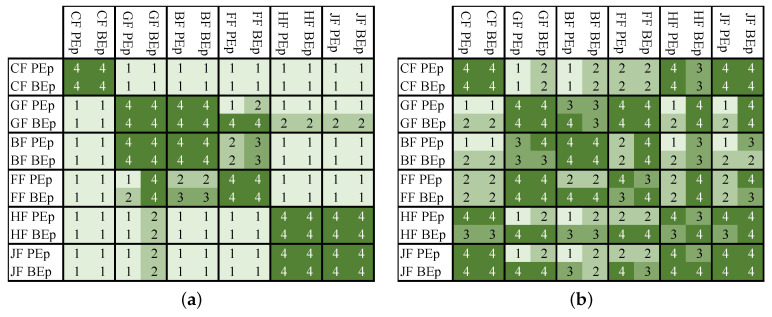
*T*-test with 95% confidence interval between all tested fibers and matrices considering (**a**) the specific stiffness and (**b**) the GWP-weighted specific stiffness. The color map follows the numbers inside the table, where 1, 2, 3, and 4 means p≤0.001, p≤0.01, p≤0.05, and p≥0.05.

A statistical analysis is performed aiming at evaluating the differences between the data shown in Figure 10. *T*-tests were performed among all sample sets and are shown in Figure 12. The confidence interval was set at 95%. The null hypothesis—statistically equal averages—is satisfied in between the sets with dark green (or number four) and rejected in all other cases (numbers from 1 to 3 and slightly lighter green). The numbers represent the level of significance, in which 1, 2, 3, and 4, means p≤0.001 (highly significant), p≤0.01 (very significant), p≤0.05 (significant), and p≥0.05 (not significant), respectively, and also varying from light to dark green.

As expected, the mechanical performance difference between the same fiber and different resins is negligible, and this is shown in the fiber blocks in Figure 12a. Furthermore, hemp and jute fibers have no statistical difference regarding the specific stiffness. One can also see that glass and basalt fibers could be grouped when being evaluated by the specific stiffness of the component. It is worth noticing that the error sources from the datasets shown in Figure 12a are standard deviations. The same cannot be said when considering Figure 12b. As already mentioned, since the GWP data from both fibers and matrices were considered as deviations of an average, the tested distributions were larger than the specific stiffness (Figure 10). This results in much more not significant differences between the averages (confirming the null hypotheses).

Another meaningful point is the applicability of these different sets of fibers and resins to the requirement of the component. The (absolute) stiffness of the carbon BB generates a maximum displacement of 1.5 mm ± 0.05 mm with a force of 500 N, which is slightly higher than the general applied force value on a rim brake [39]. By evaluating the boundary conditions (see Figure 2) along with the construction of the part and using the classical laminate theory, one could directly add up preforms, thus, summing the stiffness directly (ABD matrix with B=0 and no bending moments). The results of this evaluation are shown in Table 9.

In comparison, glass and basalt components required two more layers to get similar stiffness to CF ones. This means, naturally, a heavier component, thus more emissions. However, for these two fibers, even with more mass involved, the emissions are much lower than carbon. This changes when considering natural plant-based fibers. They required at least four double layers (eight preforms in total) to obtain similar stiffness. Observing their GWP, HF+PEp and JF+PEp perform close to or worse than CF.

## 4. Conclusions

In this publication, experiments on a part level were conducted to compare the performance of different fiber+resin systems using specific stiffness and a global warming potential (GWP)-weighted specific stiffness, i.e., a value related to ${\mathrm{CO}}_{2}^{\mathrm{eq}.}$, as the main comparison parameters. The first parameter is well known, mainly in the aerospace industry, and carbon and glass fibers are the best performers. The second parameter is introduced in this study and has as an objective a proper trade-off between mechanical and ecological properties and was explained using the example of global warming potential (GWP), which is one aspect of an LCA.

The LCA is an instrument used for the comprehensive ecological evaluation of products, estimating their resulting environmental impact. The results of the LCA should provide an answer to the question “How much impact will have a product system have on the environment?”. As expected, when contemplating only the mechanical properties, synthetic fibers perform much better than natural ones. However, when analyzing the specific stiffness along with the GWP, natural fibers become a valuable choice. Still, as observed in key results herein provided, fibers alone do not present the full picture of the GWP in a composite part, mostly in plant-based natural fibers. It is almost indifferent to the nature of the resin when considering carbon fibers and, in the opposite direction, natural fiber-based composites’ GWP is more sensitive to resin’s than fiber’s emissions. Thus, the use of natural fibers with the aim of reducing emissions is counterproductive with petroleum-based resins. Glass fibers are in the middle ground, in which both fibers and resin emissions are close to each other. Flax fibers with the biogenic epoxy have performed well among the fibers+resin systems tested. This statement refers to the impact category ’global warming potential’, measured as the ${\mathrm{CO}}_{2}^{\mathrm{eq}.}$ in fiber production, in correlation with the mechanical parameter—specific stiffness of the composite. In this study, the entire component, the brake booster, was used as the functional unit.

A comprehensive life cycle assessment according to the ISO 14040 [40] standard includes a proper scoping of the product system, deciding which activities and processes belong to the life cycle of the product that is studied. Within a comprehensive LCA, following the aforementioned ISO standard [40], a suitable framework is established for the product system. This framework includes decisions regarding the inclusion of activities and processes within the product’s life cycle for analysis. It also encompasses the incorporation of various impact assessment parameters like acidification potential (AP), eutrophication potential (EP), ozone depletion potential (ODP), land use (ecological footprint), cumulative energy demand (CED), human toxicity potential (HTP), and others. Additionally, considerations involve selecting geographical and temporal boundaries, the context of the study, relevant technology levels in the product system processes, and the study’s perspective—whether it should follow an assessment path to anticipate impacts of alternative choices or focus on attributing impacts linked to the studied activity.

These comprehensive studies are necessary in order to carry out an objective sustainability assessment of material systems and products. Due to the great complexity, which is mainly due to the difficulties in obtaining the relevant data, most publications, such as this one, contain partial aspects of a LCA. Future works will apply this framework so as to evaluate the stiffness of such components against other parameters of the LCA. Furthermore, the inclusion of a life-cycle costing evaluation, considering both synthetic and natural fibers, is also planned along with recycling aspects of both fibers and matrices. 

## Figures and Tables

**Table 1:** Properties of the fibers within the study. The values of density and linear density are taken from the manufacturer datasheets.

Fiber	Accryn.	Type	Density	Fiber	Roving Cross
Material			[g/cm^3^]	Fineness [tex]	Section [mm^2^]
Carbon	CF	Synthetic	1.78	1600	0.899
Glass	GF	Synthetic	2.54	2400	0.945
Basalt	BF	Natural-Mineral	2.70	2400	0.889
Flax	FF	Natural-Plant	1.50	1031	0.687
Hemp	HF	Natural-Plant	1.50	1250	0.833
Jute	JF	Natural-Plant	1.50	1667	1.111

**Table 2:** Properties of the resins within the study. The density values are taken from the manufacturer datasheets.

Epoxy +	Nature	Accryn.	Density	Viscosity @	Young’s Modulus
Hardener			[g/cm^3^]	20 °C [mPa·s]	[MPa]
L20 + EpH 161	Petroleum	PEp	1.158	900	2400
InfuGreen 810 + SD 8822	Plant ^†^	BEp	1.095	320	2600

^†^ 36% of the epoxy resin is plant-based [25].

**Table 3:** kg ${\mathrm{CO}}_{2}^{\mathrm{eq}.}$/kg fiber (GWP) for each fiber, along with the references from which these values were obtained.

GWP	CF	GF	BF	FF	HF	JF
$\frac{\mathrm{kg}\;{\mathrm{CO}}_{2}^{\mathrm{eq}.}}{\mathrm{kg}\;\mathrm{fiber}}$	22.4 [27]	2.03 [28]	0.398 [14]	0.349 [16]	0.406 [16]	0.548 [16]
	26.4 [13]	2.50 [16]	0.153–0.968 [15]	0.437 [29]	1.600 [29]	1.3–1.9 [30]
	31.0 [2]	2.60 [12]				

**Table 4:** kg ${\mathrm{CO}}_{2}^{\mathrm{eq}.}$/kg resin (GWP) for each resin, along with the references from which these values were obtained.

GWP	BEp	PEp
$\frac{\mathrm{kg}\;{\mathrm{CO}}_{2}^{\mathrm{eq}.}}{\mathrm{kg}\;\mathrm{resin}}$	1.42–2.85 [7]	4.68 [32]
	4.079 [11]	6.70 [11,33]

**Table 5:** GWP average values (plus their variations) considered in this study.

GWP $\left[\frac{\mathrm{kg}\;{\mathrm{CO}}_{2}^{\mathrm{eq}.}}{\mathrm{kg}\;\mathrm{material}}\right]$			
CF	26.70±4.300	GF	2.315±0.285
BF	0.561±0.408	FF	0.393±0.044
HF	1.003±0.597	JF	1.224±0.676
PEp	5.690±1.001	BEp	2.750±1.330

**Table 6:** Roving measured linear density, difference between the measured and the datasheet values, and the corrected roving cross-section.

Fiber	Fiber	Difference	Roving Cross
	Fineness [tex]	[%]	Section [mm^2^]
CF	1617.14 ± 28.00	1.07	0.908 ± 0.016
GF	2419.76 ± 20.29	0.82	0.952 ± 0.008
BF	2464.68 ± 39.30	2.69	0.913 ± 0.015
FF	963.77 ± 51.50	−6.48	0.642 ± 0.034
HF	1066.93 ± 159.32	−14.65	0.711 ± 0.106
JF	1496.02 ± 165.17	−10.24	0.997 ± 0.110

**Table 7:** Measured densities of the epoxy resins showing differences between the datasheets and the values obtained by experiment.

	Density [g/cm^3^]	Difference [%]
PEp	1.134±0.002	2.07
BEp	1.131±0.002	3.29

**Table 8:** Mass ($m^{BB}$) and fiber volume content ($f_{vc}$) of the specimens with the different fibers and resin systems. The standard deviation is calculated from five samples.

	PEp		BEp	
	$m^{\mathrm{BB}}$ [g]	$f_{\mathrm{vc}}$ [%]	$m^{\mathrm{BB}}$ [g]	$f_{\mathrm{vc}}$ [%]
CF	36.18 ± 1.30	55.05 ± 1.72	36.58 ± 1.03	54.18 ± 2.01
GF	49.22 ± 2.24	53.54 ± 4.24	50.08 ± 1.51	51.42 ± 1.70
BF	49.54 ± 1.34	48.59 ± 2.13	51.82 ± 1.51	48.06 ± 1.90
FF	31.58 ± 1.57	43.41 ± 1.94	30.45 ± 1.08	43.48 ± 1.51
HF	35.48 ± 1.67	38.48 ± 2.02	36.14 ± 0.83	38.37 ± 0.84
JF	55.78 ± 2.18	39.94 ± 1.40	55.94 ± 1.53	38.86 ± 2.80

**Table 9:** Comparison between components stiffness, masses, and GWP for a real part application. ^†^ no. layers mean double-mirrored layers (to maintain symmetry).

	CF		GF		BF		FF		HF		JF	
	PEp	BEp	PEp	BEp	PEp	BEp	PEp	BEp	PEp	BEp	PEp	BEp
u @ F [500 N]	1.442	1.414	1.418	1.504	1.530	1.422	1.427	1.374	1.393	1.364	1.23	1.272
no. layers ^†^	1		2		2		4		5		4	
Part mass [g]	36.18	36.58	98.44	100.2	99.07	103.6	126.3	121.8	177.4	180.7	223.1	223.8
$\frac{\mathrm{kg}\;{\mathrm{CO}}_{2}^{\mathrm{eq}.}}{\mathrm{part}}$	0.705	0.675	0.321	0.245	0.198	0.129	0.385	0.189	0.634	0.353	0.804	0.455

## Data Availability

Dataset available on request from the authors.

## References

[1] Hermansson F., Heimersson S., Janssen M., Svanström M. (2022). Can carbon fiber composites have a lower environmental impact than fiberglass?. Resour. Conserv. Recycl..

[2] Das S. (2011). Life cycle assessment of carbon fiber-reinforced polymer composites. Int. J. Life Cycle Assess..

[3] Spinacé M., Lambert C., Fermoselli K., Paoli M.A.D. (2009). Characterization of lignocellulosic curaua fibres. Carbohydr. Polym..

[4] Faruk O., Bledzki A., Fink H.P., Sain M. (2014). Progress Report on Natural Fiber Reinforced Composites. Macromol. Mater. Eng..

[5] Dong C. (2017). Review of natural fibre-reinforced hybrid composites. J. Reinf. Plast. Compos..

[6] Marcuello C., Chabbert B., Berzin F., Bercu N., Molinari M., Aguié-Béghin V. (2023). Influence of Surface Chemistry of Fiber and Lignocellulosic Materials on Adhesion Properties with Polybutylene Succinate at Nanoscale. Materials.

[7] Mindermann P., Pérez M.G., Knippers J., Gresser G.T. (2022). Investigation of the Fabrication Suitability, Structural Performance, and Sustainability of Natural Fibers in Coreless Filament Winding. Materials.

[8] Deeraj B., Joseph K., Jayan J., Saritha A. (2021). Dynamic mechanical performance of natural fiber reinforced composites: A brief review. Appl. Sci. Eng. Prog..

[9] Nessi S., Sinkko T., Bulgheroni C., Garcia-Gutierrez P., Giuntoli J., Konti A., Mengual E.S., Tonini D., Pant R., Marelli L. (2021). Life Cycle Assessment (LCA) of Alternative Feedstocks for Plastics Production.

[10] Marson A., Piron M., Zuliani F., Fedele A., Manzardo A. (2023). Comparative Life Cycle Assessment in the plastic sector: A systematic literature review. Clean. Environ. Syst..

[11] La Rosa A.D., Recca G., Summerscales J., Latteri A., Cozzo G., Cicala G. (2014). Bio-based versus traditional polymer composites. A life cycle assessment perspective. J. Clean. Prod..

[12] Duflou J.R., Deng Y., van Acker K., Dewulf W. (2012). Do fiber-reinforced polymer composites provide environmentally benign alternatives? A life-cycle-assessment-based study. Mater. Res. Soc. Bull..

[13] Khorgade P., Rettinger M., Burghartz A., Schlaich M. (2023). A comparative cradle-to-gate life cycle assessment of carbon fiber-reinforced polymer and steel-reinforced bridges. Struct. Concr..

[14] Fořt J., Koči J., Černý R. (2021). Environmental Efficiency Aspects of Basalt Fibers Reinforcement in Concrete Mixtures. Energies.

[15] Azrague K., Inman M.R., Alnæs L.I., Schlanbusch R.D., Jóhannesson B., Sigfusson T.I., Thorhallsson E.R., Franzson H., Arnason A.B., Vares S. Life Cycle Assessment as a tool for resource optimisation of continuous basalt fibre production in Iceland. Proceedings of the Life Cycle Assessment and Other Assessment Tools for Waste Management and Resource Optimization.

[16] De Beus N., Carus M., Barth M. (2019). Carbon Footprint and Sustainability of Different Natural Fibres for Biocomposites and Insulation Material.

[17] Niutta C.B., Ciardiello R., Tridello A., Paolino D.S. (2023). Epoxy and Bio-Based Epoxy Carbon Fiber Twill Composites: Comparison of the Quasi-Static Properties. Materials.

[18] Mattheij P., Gliesche K., Feltin D. (1998). Tailored Fiber Placement - Mechanical Properties and Applications. J. Reinf. Plast. Compos..

[19] Mattheij P., Gliesche K., Feltin D. (2000). 3D reinforced stitched carbon/epoxy laminates made by tailored fibre placement. Compos. Part Appl. Sci. Manuf..

[20] Spickenheuer A. (2014). Zur Fertigungsgerechten Auslegung von Faser-Kunststoff-Verbundbauteilen für den Extremen Leichtbau Aufbasis des Variabelaxialen Fadenablageverfahrens Tailored Fiber Placement. Ph.D. Thesis.

[21] Bittrich L., Spickenheuer A., Almeida J., Müller S., Kroll L., Heinrich G. (2019). Optimizing variable-axial fiber-reinforced composite laminates: The direct fiber path optimization concept. Math. Probl. Eng..

[22] Uhlig K., Bittrich L., Spickenheuer A., Almeida J.H.S. (2019). Waviness and fiber volume content analysis in continuous carbon fiber reinforced plastics made by tailored fiber placement. Compos. Struct..

[23] Almeida J., Bittrich L., Nomura T., Spickenheuer A. (2019). Cross-section optimization of topologically-optimized variable-axial anisotropic composite structures. Compos. Struct..

[24] Poniecka A., Barburski M., Ranz D., Cuartero J., Miralbes R. (2022). Comparison of Mechanical Properties of Composites Reinforced with Technical Embroidery, UD and Woven Fabric Made of Flax Fibers. Materials.

[25] Sicomin Epoxy Systems SR InfuGreen 810: Green Epoxy Systems for Injection and Infusion—Technical Datasheet. http://sicomin.com/datasheets/product-pdf1167.pdf.

[26] Spickenheuer A., Scheffler C., Bittrich L., Haase R., Weise D., Garray D., Heinrich G. Tailored Fiber Placement in Thermoplastic Composites. Proceedings of the 3rd International MERGE Technologies Conference (IMTC) 2017.

[27] The Japan Carbon Fiber Manufacturers Association (2022). Overview of LCI Data for Carbon Fiber. https://www.carbonfiber.gr.jp/english/tech/lci.html.

[28] PwC (2023). Life Cycle Assessment of CFGF—Continuous Filament Glass Fibre Products.

[29] González-García S., Hospido A., Feijoo G., Moreira M. (2010). Life cycle assessment of raw materials for non-wood pulp mills: Hemp and flax. Resourses Conserv. Adn Recycl..

[30] Van Dam J., Bos H. (2004). The Environmental Impact of Fibre Crops in Industrial Applications.

[31] Sicomin Epoxy Systems Eco Profil. https://greenpoxy.org/ecoprofil/.

[32] Patel M. (2003). Cumulative energy demand (CED) and cumulative CO_2_ emissions for products of the organic chemical industry. Energy.

[33] Chard J.M., Basson L., Creech G., Jesson D.A., Smith P.A. (2019). Shades of Green: Life Cycle Assessment of a Urethane Methacrylate/Unsaturated Polyester Resin System for Composite Materials. Sustentability.

[34] Konze S., Lisbôa T.V., Bittrich L., Stommel M., Spickenheuer A. Low-cost rapid-prototyping manufacturing process for tailored fiber placement components. Proceedings of the International Congress on Composite Materials.

[35] (2000). Textile Glass-Yarns-Determination of Breaking Force and Breaking Elongation.

[36] (2010). Textiles-Yarns from Packages-Determination of Single-End Breaking Force and Elongation at Break Using Constant Rate of Extension (CRE) Tester.

[37] (2019). Plastics-Methods for Determining the Density of Non-Cellular Plastics—Part 1: Immersion Method, Liquid Pycnometer Method and Titration Method.

[38] Poniecka A., Barburski M., Puszkarz A. (2023). Analysis of the effect of embroidery density on the strength of the composite containing technical embroidery made of flax fibers as reinforcement. Book of Abstracts, Proceedings of the 6th International Conference on Natural Fibers, Funchal, Portugal, 19–21 June 2023.

[39] Oertel C., Neuburger H., Sabo A. (2010). Construction of a test bench for bicycle rim and disc brakes. Procedia Eng..

[40] (2006). Environmental Management-Life Cycle Assessment—Principles and Framework.

